# First description of *Bartonella koehlerae* infection in a Spanish dog with infective endocarditis

**DOI:** 10.1186/s13071-017-2188-3

**Published:** 2017-05-19

**Authors:** María-Dolores Tabar, Laura Altet, Ricardo G. Maggi, Jaume Altimira, Xavier Roura

**Affiliations:** 1 Hospital Veterinario San Vicente, Calle del Veterinario Manuel Isidro Rodríguez García N°17, San Vicente del Raspeig, Alicante, 03690 Spain; 2 Vetgenomics, Parc de Recerca UAB Edifici Eureka, Campus de la UAB Bellaterra, 08913 Barcelona, Spain; 3 Vector Borne Disease Diagnostic Lab, North Carolina State University, 1060 William Moore Drive, Room 462ª, Raleigh, NC 27607 USA; 4 Histovet, Servicio de Diagnóstico Histopatológico Veterinario, Avda. Països Catalans 12, 12 local D, 08192 Sant Quirze del Vallés, Barcelona, Spain; 5 Hospital Clínic Veterinari, Carrer de l´Hospital, Campus UAB, Universitat Autònoma de Barcelona, 08193 Bellaterra, Barcelona, Spain

**Keywords:** *Bartonella*, Dog, Endocarditis

## Abstract

**Background:**

*Bartonella koehlerae* has been recently described as a new cat- and cat fleas-associated agent of culture-negative human endocarditis. It has been also encountered in one dog from Israel and six dogs from the USA, but other clinically relevant reports involving this bacterium are lacking.

**Results:**

A 7-year-old intact male mixed dog presented with clinico-pathological signs consistent with mitral endocarditis and cutaneous hemangiosarcoma. Molecular studies revealed the presence of *Bartonella koehlerae* DNA in samples from blood and mitral valve tissue.

**Conclusions:**

This is the first description of *B. koehlerae* in Spain, corroborating that it can also be detected in dogs. *Bartonella koehlerae* infection should also be considered in Spain in humans and dogs presenting with clinical disease suggestive of it, such as culture-negative endocarditis.

**Electronic supplementary material:**

The online version of this article (doi:10.1186/s13071-017-2188-3) contains supplementary material, which is available to authorized users.

## Background

*Bartonella* spp. are the etiological agents in humans and animals of several emergent and re-emergent vector-borne diseases that have a broad spectrum of clinical presentations including endocarditis, granulomatous diseases, meningoencephalitis, polyarthritis, uveitis or hemolytic anemia [1].

*Bartonella koehlerae* has been documented as a human pathogen and it has been increasingly detected in the last years [2, 3]. There are sporadic isolated descriptions of this bacterium in dogs. *Bartonella koehlerae* has been described in seven dogs from Israel and the USA with endocarditis, splenic disease, with suspicion of having a vector-transmitted infection or with hyperinsulinemic hypoglycemia syndrome [4–7].

## Methods

A case report of a dog with endocarditis is herein presented. Clinical investigation was performed and complemented with postmortem evaluation at Hospital Veterinario San Vicente (San Vicente del Raspeig, Alicante, Spain). Techniques employed for laboratory workup were Procyte Dx Hematology Analyzer-Idexx, Catalyst Dx Chemistry Analyzer and IDEXX VetLab® UA™. Serologies were performed with rapid tests (Test SNAP® Leishmania and test SNAP 4Dx, IDEXX). Histopathology was performed at Histovet (Servicio de Diagnóstico Histopatológico Veterinario, Sant Quirze del Vallés, Barcelona, Spain).

Molecular investigation was initially performed at the SVGM (Servei Veterinari de Genètica Molecular, Facultat de Veterinaria, Universitat Autònoma de Barcelona, Spain) with EDTA-blood samples and freshly frozen tissue samples and further investigation was completed at the Intracellular Pathogens Research Laboratory (Center for Comparative Medicine and Translational Research, College of Veterinary Medicine, North Carolina State University, Raleigh, NC, USA). Samples shipped to the Intracellular Pathogens Research Laboratory consisted on DNA previously extracted at SVGM, EDTA-blood sample and paraffin-embedded tissue.

PCR screening of *Bartonella* ITS region was performed by real-time PCR using oligonucleotides BsppITS325s: 5'-CTT CAG ATG ATG ATC CCA AGC CTT YTG GCG-3' and BsppITS543as: 5'-TAA AYT GGT GGG CCT GGG AGG ACT TG-3' as forward and reverse primers, respectively, to increase the sensitivity of the detection of a broader range of *Bartonella* species [8]. Amplification was performed in a 25-μl final volume reaction containing 12.5 μl of 2× Sso Advanced Universal Sybr Green Supermix (BioRad), 0.2 μl of 100 μM of each forward and reverse primer (IDT® DNA Technology) and 5 μl of DNA from each sample tested. PCR negative controls were prepared using 5 μl of DNA from blood of a healthy dog. Positive controls for PCR were prepared by serial dilution (using dog blood DNA) of genomic DNA from *B. henselae* down to 0.001 pg/μl (equivalent to 0.5 bacteria per μl). qPCR was performed in an BioRad CFX96 Real-Time System, under the following conditions: a single hot-start cycle at 95 °C for 2 min followed by 55 cycles of denaturing at 94 °C for 10 s, annealing at 66 °C for 10 s, and extension at 72 °C for 15 s. Amplicons products were sequenced to establish species strain identification. The sensitivity of the qPCR assay is 98% at 0.001 pg/μl (0.5 bacteria genomes per μl).

## Results

A 7-year-old intact male mixed dog was presented for a three-week history of anorexia, weight loss, polyuria and polydipsia that had worsened to severe generalized weakness at presentation time. There was also a cutaneous mass located in the right flank that had ruptured some days before resulting in serosanguinolent discharge. The dog lived outdoors, was regularly vaccinated and dewormed. Physical examination abnormalities included poor body condition (BCS, 2/9), fever (40 °C), tachycardia, grade 3-heart apex systolic murmur, pain in several joints, abdominal discomfort and an ulcerated cutaneous mass in the abdomen.

Laboratory workup revealed left shift leukocytosis (45.53 M/μl; reference range, 5.5–16.9 M/μl), neutrophilia (37.01 K/μl; reference range, 2–12 K/μl), thrombocytopenia (114 K/μl; reference range, 175–500 K/μl), azotemia (creatinine 2.1 mg/dl; reference range, 0.5–1.8 mg/dl, blood urea nitrogen, BUN 60 mg/dl; reference range, 7–27 mg/dl), increased ALP (388 U/l; reference range, 23–212 U/l), hypoalbuminemia (1.6 g/dl; reference range, 2.3–4.0 g/dl), low USG (1.020) and proteinuria (dipstick analyses ≥ 500 mg/dl). Microbiologic urine and blood cultures, and *Leishmania* and SNAP 4Dx (*Ehrlichia canis*, *Dirofilaria immitis*, *Anaplasma platys*, *Anaplasma phagocythophilum* and *Borrelia burgdorferi*) serologies were negative.

Abdominal ultrasound evidenced generalized splenomegaly and diffuse nodular hypoechoic peripheral lesions in both kidneys. Thorax radiographs showed an alveolar pattern on right lung lobes and left cranial lung lobes. Thickened mitral valve with regurgitation was detected on echocardiography. Fine needle aspiration of the cutaneous mass and spleen were consistent with a mesenchymal neoplasm and reactive splenomegaly, respectively.

**Fig. 1:**
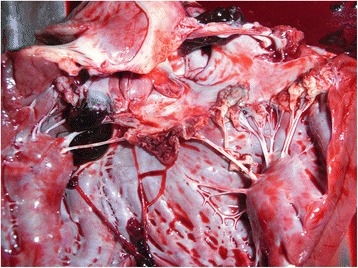
Macroscopic view of mitral valve endocarditis on necropsy

**Fig. 2:**
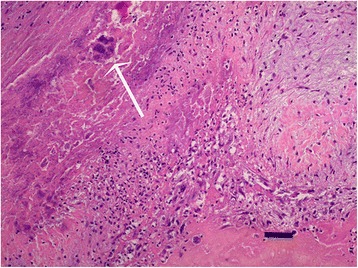
Endocardium. Mixed inflammatory and fibrinous exudate associated to a bacterial colony (*white arrow*). H&E staining (×200). *Scale-bar*: 40 μm

**Fig. 3:**
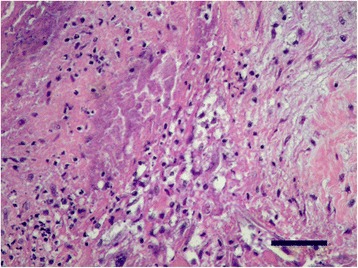
Endocardium. Fibrinous and neutrophilic exudation accompanied with intense fibrovascular reactivity (bottom right). H&E staining (×400). *Scale-bar*: 40 μm

Intensive treatment for presumptive infective endocarditis and septic shock was performed with fluid therapy, antibiotics (ampicillin and enrofloxacin), ranitidine and low molecular weight heparin. However health status deteriorated rapidly and the dog died twelve hours after admission. Necropsy confirmed mitral endocarditis (Figs. 1, 2, 3) and secondary inflammatory, necrotic, hemorrhagic and thrombotic lesions in kidneys (renal infarction, glomerulonephritis), lungs (bronchopneumonia) and spleen. Histopathology of the cutaneous mass revealed a neoplastic proliferation of endothelial cells consistent with hemangiosarcoma.

**Fig. 4:**
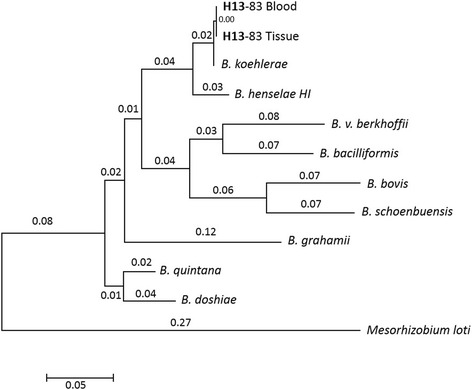
Evolutionary relationships of taxa. The evolutionary history was inferred using the Neighbor-Joining method. The optimal tree is drawn to scale, with branch lengths (next to the branches) in the same units as those of the evolutionary distances used to infer the phylogenetic tree. The evolutionary distances were computed using the Maximum Composite Likelihood method and are in the units of the number of base substitutions per site

A polymerase chain reaction (PCR) targeting a fragment of the 16S-23S ribosomal RNA intergenic spacer (ITS) was performed on extracted whole blood and mitral valve tissue (frozen tissue stored with saline) at the SVGM using conserved primers for species of the genus *Bartonella* as previously described [9], which yielded positive results. In order to ascertain the species involved whole blood samples and mitral valve paraffin-embedded tissue were shipped to the Intracellular Pathogens Research Laboratory (Center for Comparative Medicine and Translational Research, College of Veterinary Medicine, North Carolina State University, Raleigh, NC, USA). PCR from DNA obtained of *Bartonella*/alpha-Proteobacteria growth medium (BAPGM) pre-enrichment blood culture [8] and from the mitral valve tissue were positive for *Bartonella* spp. The presence of *Bartonella koehlerae* DNA was confirmed in blood (homology of 214/219 bp, 100% query cover, identity score 98%), and in tissue (homology of 217/219 bp, 100% query cover, identity score 99% with GenBank AF 312490 sequence) (Fig. 4).

## Discussion

Here, we report the first description of *Bartonella koehlerae* in Spain, as the causative agent of *Bartonella* endocarditis involving the mitral valve in a dog.

Infective endocarditis is an uncommon and often deadly disease in dogs that represents a diagnostic challenge in most of the cases because echocardiogram can be normal, and blood cultures can yield false-negative results due to the low-grade and intermittent bacteremia associated to *Bartonella* infection at the time of blood collection. Previous research has emphasized that negative blood cultures are especially frequent in *Bartonella*-induced endocarditis and other techniques are needed for diagnosis [10]. The combination of a pre-enrichment liquid culture with PCR amplification enhances the detection of *Bartonella* species [8]. Previous studies have shown that *B. vinsonii berkhoffii* is one of the most common agents found in canine *Bartonella* endocarditis, where infection often involves the aortic valve [10]. However other species, such as *B. koehlerae* and *B. henselae*, have been recently implicated in canine endocarditis in the USA and Israel [4, 11]. In Spain, several cases of endocarditis due to *Bartonella* spp. has been reported in people in the past ten years and the number of cases is likely to be underestimated [12].

Interestingly, recent reports suggest that *Bartonella* spp*.* should be investigated as a cause of vasoproliferative tumors in both humans and dogs, due to their ability of enhancing endothelial cell proliferation in conjunction with inhibition of apoptosis [13, 14]. Species of *Bartonella* involved are *B. bacilliformis*, *B. quintana*, *B. henselae*, *B. vinsonii berkhoffi* and *B. koehlerae*. Unfortunately, definitive association of *Bartonella* and the skin hemangiosarcoma could not be established in this study since *Bartonella* DNA could not be amplified from the skin tumor. False-negative PCR results could be caused by prolonged period of formalin fixation [15, 16]. Further research is needed to test the potential role of the genus *Bartonella* as cofactor in the development of cancer [13, 14].

In Spanish dogs, presence of canine bartonellosis has been demonstrated with serological studies [17]. Moreover, there is molecular evidence of this infection in a recently described dog with generalised pyogranulomatous disease and hyperviscosity syndrome [18]. Several reports have confirmed that *Bartonella* infection is present in people in the Valencian Community and Catalonia (Spain) [19, 20].

Definitive documentation of the causative *Bartonella* spp. in dogs may prove to be a legitimate medical concern, as dogs have been also implicated in the transmission of bartonellosis following a bite or a scratch [21, 22]. *Bartonella koehlerae* has been described as a cat-associated agent of culture-negative human endocarditis [3], and this case report corroborates that it can also be detected in dogs.

The findings presented in this manuscript reinforce the concept of “One Health” approach, which requires close communication in all aspects of health care for humans and animals, with the aim of supporting public health in general. It is worth highlighting that there are many vectors that could harbor *Bartonella* organisms as sand flies, human body lice, cat fleas, ear mites, and ticks have been described as potential vectors [1]. Therefore the risk of both animal and human exposure to *Bartonella* spp. may be more substantial than is currently believed and, until an effective vaccine in the protection against *Bartonella* spp. could be available, ectoparasites control measures should be implemented in both dogs and cats to avoid the potential of zoonotic transmission of this severe infection to humans.

## Conclusions

To our knowledge, this is the first description of *Bartonella koehlerae* in Spain, corroborating that the parasite can also be detected in dogs. *Bartonella* spp. infection should also be considered in Spain in humans and dogs presenting with features suggestive of it, such as blood culture-negative endocarditis.

## Additional file

Additional file 1: Figure S1. Alignment of the ITS region sequences comparing the dog sequences with *Bartonella henselae* and *B. koehlerae*. (TIFF 2949 kb)

## References

[1] Breitschwerdt EB, Maggi RG, Chomel BB, Lappin MR (2010). Bartonellosis: an emerging infectious disease of zoonotic importance to animals and human beings. J Vet Emerg Crit Care (San Antonio).

[2] Breitschwerdt EB, Maggi RG, Robert Mozayeni B, Hegarty BC, Bradley JM, Mascarelli PE (2010). PCR amplification of *Bartonella koehlerae* from human blood and enrichment blood cultures. Parasit Vectors.

[3] Avidor B, Graidy M, Efrat G, Leibowitz C, Shapira G, Schattner A (2004). *Bartonella koehlerae,* a new cat-associated agent of culture-negative human endocarditis. J Clin Microbiol.

[4] Ohad DG, Morick D, Avidor B, Harrus S (2010). Molecular detection of *Bartonella henselae* and *Bartonella koehlerae* from aortic valves of boxer dogs with infective endocarditis. Vet Microbiol.

[5] Varanat M, Maggi RG, Linder KE, Breitschwerdt EB (2011). Molecular prevalence of *Bartonella*, *Babesia*, and hemotropic *Mycoplasma* sp. in dogs with splenic disease. J Vet Intern Med.

[6] Pérez C, Maggi RG, Diniz PPV, Breitschwerdt EB (2011). Molecular and serological diagnosis of *Bartonella* infection in 61 dogs from the United States. J Vet Intern Med..

[7] Breitschwerdt EB, Goldkamp C, Castleman WL, Cullen JM, Mascarelli PE, Thalhem L (2014). Hyperinsulinemic hypoglicemia syndrome in 2 dogs with bartonellosis. J Vet Intern Med.

[8] Duncan AW, Maggi RG, Breitschwerdt EB (2007). A combined approach for the enhanced detection and isolation of *Bartonella* species in dog blood samples: pre-enrichment liquid culture followed by PCR and subculture onto agar plates. J Microbiol Methods.

[9] Tabar MD, Francino O, Altet L, Sánchez A, Ferrer L, Roura X (2009). PCR survey of vectorborne pathogens in dogs living in and around Barcelona, an area endemic for leishmaniosis. Vet Rec.

[10] MacDonald KA, Chomel BB, Kittleson MD, Kasten RW, Thomas WP, Pesavento P (2004). A prospective study of canine infective endocarditis in northern California (1999–2001): emergence of *Bartonella* as a prevalent etiologic agent. J Vet Intern Med.

[11] Fenimore A, Varanat M, Maggi R, Schultheiss P, Breitschwerdt E, Lappin MR (2011). *Bartonella* spp. DNA in cardiac tissues from dogs in Colorado and Wyoming. J Vet Intern Med.

[12] Oteo JA, Castilla A, Arosey A, Blanco JR, Ibarra V, Morano LE (2006). Endocarditis due to *Bartonella* spp. Three new clinical cases and Spanish literature review. Enferm Infecc y Microbiol Clin.

[13] Breitschwerdt EB, Maggi RG, Varanat M, Linder KE, Weinberg G (2009). Isolation of *Bartonella vinsonii* subsp. *berkhoffii* Genotype II from a boy with epitheloid hemangiendothelioma and a dog with hemangiopericytoma. J Clin Microbiol.

[14] Beerlage C, Varanat M, Linder K, Maggi RG, Cooley J, Kempf VA (2012). *Bartonella vinsonii* subsp*. berkhoffii* and *Bartonella henselae* as potential causes of proliferative vascular diseases in animals. Med Microbiol Immunol.

[15] Müller N, Zimmermann V, Forster U, Bienz M, Gottstein B, Welle M (2003). PCR-based detection of canine *Leishmania* infections in formalin-fixed and paraffin embedded skin biopsies: elaboration of a protocol for quality assessment of the diagnostic amplification reaction. Vet Parasitol.

[16] Turashvili G, Yang W, McKinney S, Kalloger S, Gale N, Ng Y (2012). Nucleic acid quantity and quality from paraffin blocks: defining optimal fixation, processing and DNA/RNA extraction techniques. Exp Mol Pathol.

[17] Roura X, Breitschwerdt EB, Lloret A, Ferrer L, Hegarty B (2005). Serological evidence of exposure to *Rickettsia*, *Bartonella*, and *Ehrlichia* species in healthy or *Leishmania* infected dogs from Barcelona, Spain. Intern J Appl Res Vet Med.

[18] Tabar MD, Maggi RG, Altet L, Vilafranca M, Francino O, Roura X (2011). Gammopathy in a Spanish dog infected with *Bartonella henselae*. J Small Anim Pract.

[19] Fernández-Arias C, Borrás-Máñez M, Colomina-Rodríguez J, Cuenca-Torres M, Guerrero-Espejo A (2015). Incidence of *Bartonella henselae* infection during the period 2009–2012 in the Valencian Community, Spain. Rev Esp Salud Publica.

[20] Sanfeliu I, Antón E, Pineda V, Pons I, Pérez J, Font B (2009). Description of *Bartonella* spp. infections in a general hospital of Catalonia, Spain. Clin Microbiol Infect.

[21] Tsukahara M, Tsuneoka H, Lino H, Ohno K, Murano I (1998). *Bartonella henselae* infection from a dog. Lancet.

[22] Keret D, Giladim M, Kletter Y, Wientroub S (1998). Cat-scratch disease osteomyelitis from a dog scratch. J Bone Joint Surg Br.

