# Supplementary figures and images for: First description of Bartonella koehlerae infection in a Spanish dog with infective endocarditis
Source: Parasit Vectors. 2017 May 19;10:247. doi: 10.1186/s13071-017-2188-3 (PMC5437684; doi:10.1186/s13071-017-2188-3)

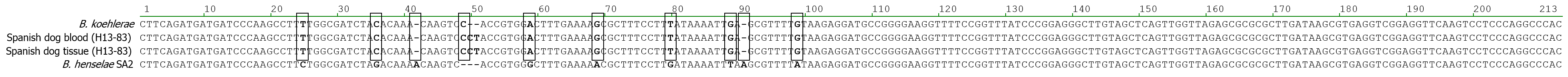

Supplement: Additional file 1: Figure S1. — Alignment of the ITS region sequences comparing the dog sequences with Bartonella henselae and B. koehlerae. (TIFF 2949 kb) [file 13071_2017_2188_MOESM1_ESM.tiff]
